# Erythema Induratum of Bazin Associated With a Negative Tuberculin Test: A Case Report

**DOI:** 10.7759/cureus.113542

**Published:** 2026-07-28

**Authors:** Valeria Gonzalez Quiroz, Sergio Eduardo Arroyo Jaramillo, Guadalupe de los Angeles Salazar Gonzalez, Jorge Alberto Garay Hernandez, Cesar Fabian Vallejo Rico, Libia Eugenia Corona Caloca, Alicia Marali Ibarra Moncivais

**Affiliations:** 1 Internal Medicine, Mexican Social Security Institute, Durango, MEX; 2 Dermatology, Mexican Social Security Institute, Durango, MEX

**Keywords:** erythema induratum of bazin, lobular panniculitis, mycobacterium tuberculosis, skin ulcer, tuberculin test

## Abstract

Bazin's indurated erythema is a chronic lobular panniculitis associated with a hypersensitivity reaction to *Mycobacterium tuberculosis*. It is characterized by recurrent painful subcutaneous nodules and ulcerative lesions on the lower extremities. We present a 39-year-old male with a one-year history of unintentional weight loss and intermittent evening fever. During this period, the patient developed progressively evolving erythematous cutaneous lesions located on the lower extremities, along with multiple painful, indurated subcutaneous nodules exhibiting a chronic recurrent course with occasional ulceration. Histopathological evaluation of a skin biopsy revealed granulomatous lobular panniculitis consistent with erythema induratum of Bazin. It is a rare tuberculid presentation, representing a cutaneous manifestation of hypersensitivity to tuberculosis, and is more frequently observed in women. It presents a diagnostic challenge due to its similarity to other inflammatory and infectious dermatoses. The diagnosis is clinical and histopathological, with findings typically demonstrating granulomatous lobular panniculitis and vasculitis. This case highlights the early recognition in tuberculosis-endemic regions, as early initiation of antituberculosis treatment is associated with significant clinical improvement.

## Introduction

Bazin's indurated erythema was described in 1861 by the French dermatologist Ernest Bazin, who observed the recurrent appearance of painful nodules on the legs of young women with a history of lymph node tuberculosis. In the 20th century, the concept of tuberculid emerged, defining a group of dermatoses considered hypersensitivity reactions to *Mycobacterium tuberculosis* antigens [1]. Its incidence is unknown due to its rarity and the lack of epidemiological records. It is considered an uncommon manifestation within the spectrum of cutaneous tuberculosis, representing only a small percentage of extrapulmonary cases. In Mexico, although the overall incidence is low, it should be considered in the differential diagnosis of chronic nodular panniculitis, given that tuberculosis remains endemic in the country. It has a higher incidence in women between 30 and 40 years of age, but can occur in any age group [2].

Clinically, it manifests as the recurrent appearance of subcutaneous nodules located primarily on the lower extremities. The lesions are most common on the posterior aspect of the legs, although they are also seen on the lateral aspects and, less frequently, on the thighs or ankles. They are usually bilateral and sometimes symmetrical [3]. The nodules develop progressively, measuring between 1 and 5 cm in diameter. On physical examination, they appear as deep, firm, and poorly defined nodules, fixed to the underlying subcutaneous tissue and painful to palpation. The skin initially shows erythema and hyperemia, which progress to ulceration as a consequence of the associated vasculitis and necrosis of adipose tissue. The ulcers are usually moderately deep, with irregular borders and a necrotic base, and may leave skin atrophy, fibrosis, or post-inflammatory hyperpigmentation [2]. Histopathologically, lobular panniculitis with fat necrosis and vasculitis of medium and small vessels is observed [4]. The diagnosis is clinicopathological and requires correlation of clinical, histopathological, and molecular findings. Detection of *M. tuberculosis *DNA by polymerase chain reaction(PCR) is a valuable diagnostic tool that strongly supports the diagnosis, although final confirmation depends on thorough clinicopathological correlation [5]. Patients usually have a favorable response to antituberculosis treatment [6].

## Case presentation

We present the case of a 39-year-old man with a history of type 2 diabetes and hypertension who experienced an unintentional weight loss of approximately 45 kg over the past year and intermittent episodes of fever, predominantly nocturnal. Subsequently, he developed progressively worsening, painful, erythematous skin lesions, mainly on his lower extremities (Figure 1). Physical examination revealed bilateral inguinal lymphadenopathy. On the posterior aspect of both legs, multiple lesions ranging from 2 to 10 cm in diameter were observed, consisting of erythematous plaques and nodules associated with edema and increased local temperature. Several of these lesions showed poorly defined central ulceration and eschar remnants. An extensive erythematous area was also observed on the left leg, with signs suggestive of incipient necrosis. Multiple deep subcutaneous nodules consistent with panniculitis were also identified. In the lower right extremity, in the posterior region of the knee, an extensive ulcer was observed measuring approximately 15 × 10 centimeters, with irregular contours and poorly defined edges, exposing the subcutaneous tissue and deeper planes (Figure 2).

**Figure 1:**
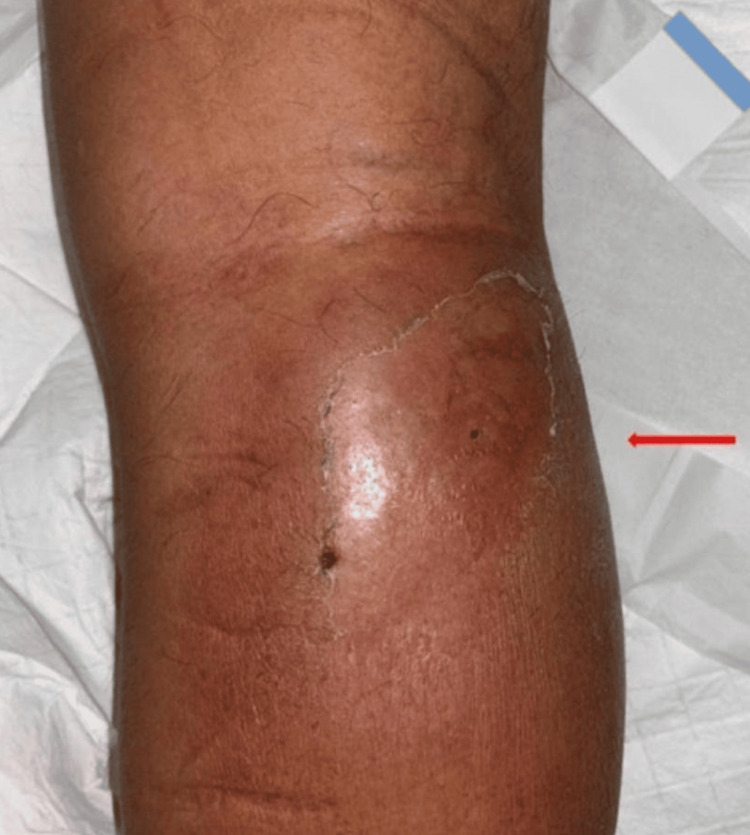
Erythematous subcutaneous nodular plaque with poorly defined borders, smooth and shiny surface with local volume increase. Anterior aspect of the leg with a subcutaneous erythematous nodular plaque with poorly defined borders, a smooth and shiny surface, and local swelling. The overlying skin is taut and shiny, with surrounding induration and slight scaling at the margins. The red arrow indicates the area of ​​greatest swelling and inflammation.

**Figure 2:**
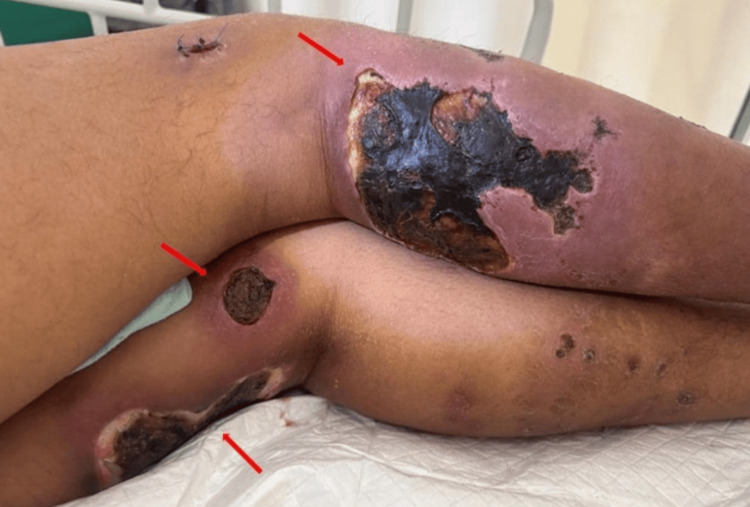
Lower extremities with multiple necrotic ulcers of irregular sizes and shapes. Extensive ulcerated plaque on the posterior aspect of the right leg, covered by a necrotic eschar with irregular and raised borders, surrounded by an erythematous-violaceous halo and perilesional induration. Smaller satellite ulcers with hemorrhagic and necrotic crusts are also observed.

Laboratory tests revealed moderate normocytic normochromic anemia, hypoalbuminemia, and reactive thrombocytosis. Serologic tests for human immunodeficiency virus (HIV), hepatitis B virus, hepatitis C virus, and VDRL were negative (Table 1). A chest computed tomography scan showed a calcified granuloma at the right lower lobe, a finding suggestive of previous granulomatous disease (Figure 3).

**Table 1:** Laboratory studies.

Variables	Results	Units	Normal parameters
Leukocytes	6.82	X 1013/L	(4-11)
Erythrocytes	2.69	X 1012/L	(4-5)
Hemoglobin	7.6	g/dL	(12-17)
Hematocrit	23	%	(35-52)
Mean corpuscular volume	85.5	fL	(80-100)
Mean corpuscular hemoglobin	28.3	pg/cell	(27-31.2)
Platelets	429	X 103/mm^3^	(130-400)
Neutrophils #	3.86	X 103/mm^3^	(2-8)
Lymphocytes #	2.08	X 103/mm^3^	(1-5)
Eosinophils #	0.1	X 103/mm^3^	(0-0.40)
Basophils #	0.3	X 103/mm^3^	(0-0.20)
Glucose	80	mg/dL	(74-106)
Urea	10	mg/dL	(19-43)
Urea nitrogen	4.6	mg/dL	(9.0-20)
Creatinine	0.5	mg/dL	(0.60-1.20)
Total proteins	7.1	g/dL	(6.3-8.2)
Albumin	2.5	g/dL	(3.5-5)
Total bilirubin	0.6	mg/dL	(0.2-1.3)
Indirect bilirubin	0.4	mg/dL	(0-1.1)
Direct bilirubin	0.2	mg/dL	(0-1)
Aspartate aminotransferase	17	U/L	(14-36)
Alanine aminotransferase	20	U/L	(35)
Alkaline phosphatase	100	U/L	(38-126)
Lactate dehydrogenase	127	U/L	(120-246)
Sodium	138	mmol/L	(137-145)
Potassium	4.4	mmol/L	(3.5-5.1)
Serum chloride	106	mmol/L	(98-107)
Calcium	8.5	mg/dL	(8.4-10.2)
Phosphorus	4	mg/dL	(2.5-4.5)
Magnessium	1.6	mg/dL	(1.6-2.4)
Prothrombin time	15	Seconds	(11.9-15.6)
Prothrombin time witness	13.5	Seconds	
International standardized index	1.21		
Partial thromboplastin time	36	Seconds	(32.22-40.14)
Partial thromboplastin time index	36.18	Seconds	
HBV surface antigen	Non-reactive	Non-reactive	Negative
Anti-HCV antibody	Non-reactive	Non-reactive	Negative
Anti-HIV antibody	Non-reactive	Non-reactive	Negative
VDRL	Non-reactive	Non-reactive	Negative

**Figure 3:**
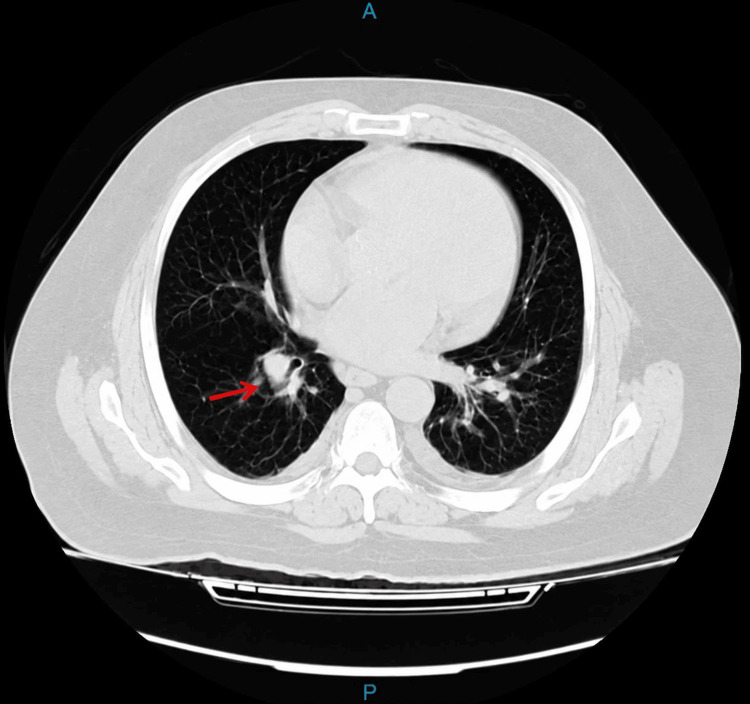
Axial computed tomography of the chest showing a calcified pulmonary granuloma in the right lower lobe (yellow arrow). Axial computed tomography of the chest showing a calcified pulmonary granuloma in the right lower lobe (yellow arrow).

Given the suspicion of panniculitis associated with a chronic infectious process, a tuberculin skin test (TST/PPD) was performed, which yielded a negative result with 2 mm of induration. Subsequently, a deep excisional biopsy was performed on one of the lesions of the lower extremity (Figure 4). The histopathological study revealed a predominantly lobular panniculitis characterized by marked fat necrosis and a dense inflammatory infiltrate. Of particular note was the presence of prominent granulomatous inflammation with epithelioid histiocytes and Langhans giant cells, along with extensive vasculitis affecting medium- and small-sized vessels, which supported the diagnosis of Bazin's erythema induratum. Furthermore, the polymerase chain reaction (PCR) performed on skin tissue was positive for *M. tuberculosis.*

**Figure 4:**
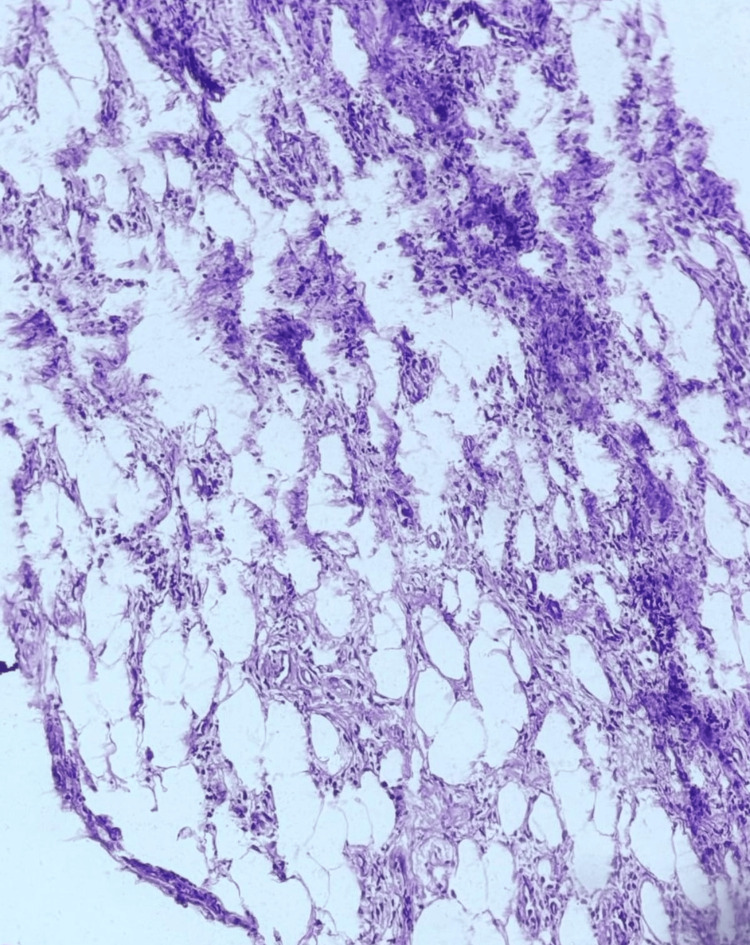
Histopathological study of subcutaneous tissue stained with hematoxylin-eosin (H&E), showing a pattern of lobular panniculitis, characterized by predominant inflammatory infiltrate in the adipose tissue lobules, with relative preservation of the interlobular septa and vasculitis (original magnifications ×100). Subcutaneous adipose tissue was observed with a predominantly lobular inflammatory infiltrate and alteration of the normal adipose architecture. The inflammatory infiltrate was composed mainly of mononuclear inflammatory cells and histiocytes, with focal areas of adipocyte degeneration and fat necrosis. Associated fibrotic and inflammatory changes were observed in the subcutaneous tissue, along with vasculitis consistent with lobular panniculitis.

During hospitalization, the patient developed a secondary bacterial superinfection of the skin lesions, with *Acinetobacter baumannii* being isolated in the microbiological cultures. Consequently, specific antibiotic treatment and surgical debridement were required. Given the clinical and histopathological findings, together with the positive molecular test for *M. tuberculosis*, the patient was treated with standard first-line antituberculous therapy consisting of an intensive phase with rifampicin, isoniazid, pyrazinamide, and ethambutol (two months), followed by a continuation phase with rifampicin and isoniazid (four months), for a total treatment duration of six months. The patient showed a favorable clinical response, with progressive resolution of the inflammatory lesions and no evidence of disease progression or recurrence during follow-up.

## Discussion

Indurated erythema of Bazin is an uncommon manifestation of cutaneous tuberculosis that remains a diagnostic challenge due to its low prevalence and heterogeneous presentation [3]. The clinical presentation in this case aligns with the classic manifestations of the disease, which pathophysiologically involves a type IV hypersensitivity reaction to *M. tuberculosis *antigens within the subcutaneous fat, leading to granulomatous lobular panniculitis and secondary necrotizing vasculitis.

A key feature of this case is the negative TST/PPD result, which serves as the main distinguishing and challenging aspect of the diagnosis. In the context of confirmed *M. tuberculosis *DNA via PCR, a false-negative TST can be attributed to several underlying mechanisms. Immunological anergy is a well-documented phenomenon where the host's cell-mediated immune response is transiently suppressed. This status can be secondary to comorbidities such as poorly controlled diabetes mellitus, which impairs T-cell function and chemotaxis, or nutritional deficiencies. Furthermore, an overwhelming systemic infection or a high bacterial load can paradoxically induce peripheral immune tolerance or energy, rendering the skin test non-reactive. Technical factors, including improper antigen storage or intradermal injection errors, must also be considered in the differential analysis of non-reactivity. Additionally, a notable epidemiological feature of this case is that, unlike the historical trends in the literature, which predominantly report adult female predominance, this case underscores the occurrence of the disease in a young male patient.

The differential diagnosis of Bazin's indurated erythema includes various inflammatory, infectious, vascular, and neoplastic panniculitis. The most important clinical differential diagnosis is erythema nodosum, which typically presents as painful, non-ulcerated nodules on the anterior aspect of the legs and is histologically characterized by septal panniculitis without vasculitis. In contrast, Bazin's indurated erythema generally manifests as recurrent, painful nodules on the posterior aspect of the legs, which may ulcerate and scar. Histopathology usually shows lobular panniculitis with granulomatous inflammation and vasculitis.

Other relevant differential diagnoses include nodular vasculitis unrelated to tuberculosis, subcutaneous sarcoidosis, lupus panniculitis, panniculitis associated with alpha-1 antitrypsin deficiency, pancreatic panniculitis, infectious panniculitis caused by atypical mycobacteria or fungi, and subcutaneous panniculitis-like T-cell lymphoma. Therefore, the diagnosis of Bazin's erythema induratum requires correlating the clinical presentation with the findings of a deep skin biopsy and microbiological or molecular evidence of *M. tuberculosis*, when available [7].

The B symptoms and the finding of a calcified pulmonary granuloma on chest computed tomography were indicative of a possible infectious etiology due to tuberculosis. The diagnosis was confirmed by histopathological examination, which identified lobular panniculitis accompanied by prominent granulomatous inflammation and secondary vasculitis, consistent with previous descriptions. Furthermore, a positive PCR test for *M. tuberculosis *on skin tissue established the definitive link between the lesions and the infection. While conventional diagnostic methods, such as Ziehl-Neelsen staining and mycobacterial cultures, often exhibit low sensitivity and high false-negative rates in cutaneous tuberculosis due to the pauci-bacillary nature of the disease, PCR offers a high degree of specificity and rapid detection of bacterial DNA, overcoming the limitations of standard microbiological techniques. However, PCR results must always be interpreted in conjunction with clinical and histopathological findings, as it detects DNA fragments rather than viable organisms. Following the initiation of antituberculosis treatment, a progressive decrease in inflammation and a reduction in the size of the lesions were observed until their complete resolution. 

Literature review and comparison with previously reported cases

To contextualize the findings of this case, a comprehensive literature review was conducted in the PubMed and ScienceDirect databases using combinations of keywords such as "Bazin's indurated erythema," "cutaneous tuberculosis," "panniculitis," and "negative tuberculin skin test." For the comparative analysis, only cases with detailed clinical presentation, diagnostic confirmation through molecular or histopathological findings, and therapeutic outcomes published in English or Spanish were selected (Table 2). 

**Table 2:** Comparison of previously reported cases of Erythema induratum of Bazin with the present case.

Year	Country	Patient	Main Clinical Findings	Tuberculosis Confirmation	Treatment
2011 [8]	Spain	73-year-old woman	Recurrent tender ulcerated nodules on the lower legs for 2 years	Histopathology and positive tuberculin skin test	Standard anti-tuberculosis therapy
2014 [9]	Mexico	10-year-old girl	Painful erythematous nodules with recurrent ulceration on the lower extremities for one year	Strongly positive tuberculin skin test and positive Mycobacterium tuberculosis PCR	Anti-tuberculosis therapy with complete clinical resolution
2021 [10]	China	16-year-old female	Erythema induratum of Bazin as the initial manifestation of cavitary pulmonary tuberculosis	Positive T-SPOT.TB assay and chest CT findings consistent with pulmonary tuberculosis	Six-month anti-tuberculosis regimen
2025 [11]	Brazil	70-year-old woman	Ulcerated indurated plaques on the lower limbs in an immunosuppressed patient	Granulomatous panniculitis on histopathology with acid-fast bacilli detected	Standard anti-tuberculosis treatment
2025 [12]	Brazil	17-year-old adolescent	Chronic atypical crusted plaques and ulceration for three years	Histopathology compatible with erythema induratum and epidemiologic evidence of tuberculosis	Anti-tuberculosis therapy with complete remission
2025 [13]	India	Elderly diabetic man	Bilateral recurrent ulcerated plaques on the lower legs associated with latent tuberculosis	Positive latent tuberculosis screening	Anti-tuberculosis therapy with marked clinical improvement
Present case	Mexico	39-year-old man	Weight loss, intermittent episodes of fever, erythematous plaques, and nodules and recurrent ulcerated plaques on the lower legs	Negative tuberculin skin test, positive Mycobacterium tuberculosis PCR, and histopathology compatible with erythema induratum	Anti-tuberculosis therapy with complete remission

## Conclusions

Indurated erythema of Bazin remains a rare manifestation of cutaneous tuberculosis and presents a significant diagnostic challenge due to its nonspecific clinical presentation and the low sensitivity of conventional diagnostic tests. In tuberculosis-endemic regions, it should be considered in the differential diagnosis of chronic nodular or ulcerative panniculitis, even in the absence of active pulmonary disease or a negative TST. This case highlights the importance of a multidisciplinary diagnostic approach that integrates clinical features, histopathological findings, and molecular techniques. The establishment of the definitive diagnosis relied on a comprehensive clinicopathological correlation supported by molecular findings, rather than by PCR alone, highlighting that the identification of *M. tuberculosis* DNA was crucial in confirming the etiology despite a non-reactive TST.

Negative reactions to the TST can be attributed to underlying immunological anergy, immunosuppression, early-stage tuberculous infection, technical limitations, or, less frequently, variations in patients with otherwise preserved cell-mediated immunity. It has been suggested that subtle immunological variability or alternative pathways in the host's T-cell receptor repertoire may lead to an insufficient localized cellular response to the purified protein derivative, which could explain the non-reactivity observed in atypical presentations. Timely antituberculosis treatment was associated with a favorable outcome, underscoring the importance of recognizing this uncommon cutaneous manifestation of tuberculosis infection and highlighting the value of molecular testing in atypical and paucibacillary forms of cutaneous tuberculosis. Increased awareness of this rare entity among clinicians can facilitate earlier diagnosis and appropriate management, particularly in countries where tuberculosis remains endemic.
